# Meniscal Replacement With a Silk Fibroin Scaffold Reduces Contact Stresses in the Human Knee

**DOI:** 10.1002/jor.24437

**Published:** 2019-08-26

**Authors:** Svenja Stein, Sabrina Höse, Daniela Warnecke, Cristina Gentilini, Nick Skaer, Robert Walker, Oliver Kessler, Anita Ignatius, Lutz Dürselen

**Affiliations:** ^1^ Institute of Orthopaedic Research and Biomechanics, Centre for Trauma Research Ulm Ulm University Medical Centre Helmholtzstraße 14 89081 Ulm Germany; ^2^ Orthox Ltd. 66 Innovation Drive, Milton Park Abingdon OX14 4RQ United Kingdom; ^3^ Centre of Orthopaedics and Sports (affiliated to Orthopaedic University Hospital, Leipziger Straße 44, 39120 Magdeburg, Germany) Albisriederstraße 243A 8047 Zurich Switzerland

**Keywords:** meniscus injury, knee joint degeneration, meniscus replacement, silk fibroin scaffold, tibiofemoral contact mechanics

## Abstract

The aim of the current study was to verify if a previously developed silk fibroin scaffold for meniscal replacement is able to restore the physiological distribution of contact pressure (CP) over the articulating surfaces in the human knee joint, thereby reducing peak loads occurring after partial meniscectomy. The pressure distribution on the medial tibial articular surface of seven human cadaveric knee joints was analysed under continuous flexion–extension movements and under physiological loads up to 2,500 N at different flexion angles. Contact area (CA), maximum tibiofemoral CP, maximum pressure under the meniscus and the pressure distribution were analysed for the intact meniscus, after partial meniscectomy as well as after partial medial meniscal replacement using the silk fibroin scaffold. Implantation of the silk fibroin scaffold considerably improved tibiofemoral contact mechanics after partial medial meniscectomy. While the reduced CA after meniscectomy was not fully restored by the silk fibroin scaffold, clinically relevant peak pressures on the articular cartilage surface occurring after partial meniscectomy were significantly reduced. Nevertheless, at high flexion angles static testing demonstrated that normal pressure distribution comparable to the intact meniscus could not be fully achieved. The current study demonstrates that the silk fibroin implant possesses attributes that significantly improve tibiofemoral CPs within the knee joint following partial meniscectomy. However, the failure to fully recapitulate the CAs and pressures observed in the intact meniscus, particularly at high flexion angles, indicates that the implant's biomechanical properties may require further improvement to completely restore tibiofemoral contact mechanics. © 2019 The Authors. *Journal of Orthopaedic Research* ^®^ published by Wiley Periodicals, Inc. on behalf of Orthopaedic Research Society. J Orthop Res 37:2583–2592, 2019

## INTRODUCTION

The crucial role of the fibrocartilaginous menisci in the structural and functional integrity of the human knee joint is well established. By increasing the articulating surface between femur and tibia as well as by converting axial loads into circumferential hoop (tensile) stresses, the menisci provide optimum load bearing inside the joint and ensure protection of the articular surfaces.^1^, ^2^, ^3^, ^4^ It was shown that peak forces in the knee joint can increase by up to six‐fold body weight during daily activities, for example, climbing stairs.^5^ Thereby, up to 81% of the tibio‐femoral joint contact force are transferred through the menisci.^6^ Consequently, meniscal tissue is especially prone to injury and meniscal lesions are the second most common knee joint injury.^7^ Irreparable lesions in the avascular area of the meniscus are usually treated by partial meniscectomy, combining the advantages of rapid pain relief and short rehabilitation in the immediate post‐operative period. However, it is well established that meniscectomy leads to osteoarthritis in the long term as the load bearing mechanism of the knee joint is severely disturbed.^8^, ^9^, ^10^ Thereby, the amount of resected meniscal tissue is directly related to the increase in contact pressure (CP) due to a reduced contact area (CA) between femur and tibia.^11^, ^12^ The more meniscal tissue is resected, the higher is the increase in CP on the articular surfaces. However, alternative treatment strategies for irreparable lesions, by which meniscal function could be restored, are limited. Despite extensive research in this field, only two scaffolds for partial meniscal replacement are clinically available, while several materials are currently under investigation.^13^ Considering the biomechanical function of the meniscus, the mechanical properties of a repair material are of major importance in the development of meniscal replacement devices.^13^, ^14^ It was previously stated, that mechanical properties should resemble native meniscal tissue as closely as possible to replace native tissue adequately.^13^ Furthermore, appropriate geometry and fixation of the device are necessary for the successful restoration of the physiological pressure distribution on the tibial plateau and to achieve long‐term chondroprotection.^13^, ^15^

A silk fibroin scaffold (FibroFix™; Orthox Ltd., Abingdon, UK) was previously developed and investigated in several in vitro and in vivo studies.^16^, ^17^, ^18^ The in vivo performance of the first generation of silk fibroin scaffolds was evaluated in a partial meniscal defect in the ovine model.^16^ Although there were encouraging results regarding biocompatibility and chondroprotection, surgical fixation of the devices required improvement. During a subsequent optimization process, a fiber mesh was integrated into the implant's porous matrix. The mechanical properties of this second‐generation silk fibroin scaffold were recently evaluated in vitro by Warnecke et al.^18^ displaying an equilibrium modulus of 0.56 ± 0.31 MPa. As a permanent implant, the silk fibroin scaffold demonstrates high mechanical competence prior to implantation, regardless of subsequent tissue ingrowth.

To assess the biomechanical functionality of the silk fibroin scaffolds in vitro, the current study was conducted, investigating tibiofemoral contact mechanics after partial meniscal replacement in human cadaveric knee joints. We postulated the hypothesis that partial meniscal replacement using a silk fibroin scaffold restores CA and CP to values of the intact knee.

## MATERIAL AND METHODS

### Study Design

Pressure distribution on the tibial articular surface of seven human knee joints was analysed in a knee joint simulator under continuous flexion and extension movements^19^ as well as in a standard materials testing machine (Zwick Z010; Zwick/Roell GmbH & Co.KG, Ulm, Germany) under physiological loads of up to 2,500 N at different flexion angles. Each knee joint was consecutively tested in three conditions:

(1) Intact medial meniscus.
(2) Partial medial meniscectomy leaving a 4 mm peripheral meniscal rim and both meniscal horns intact. This corresponds to a typical extent of meniscal resection performed in case of a vertically oriented longitudinal meniscal lesion.
(3) Partial meniscal replacement using a silk fibroin scaffold (FibroFix™).

CA, maximum pressure on the tibiofemoral CA (CP_max___Cartilage_), maximum pressure on the meniscotibial CA (CP_max_Meniscus_) and the pressure distribution were analysed.

### Silk Fibroin Scaffold

**Figure 1:**
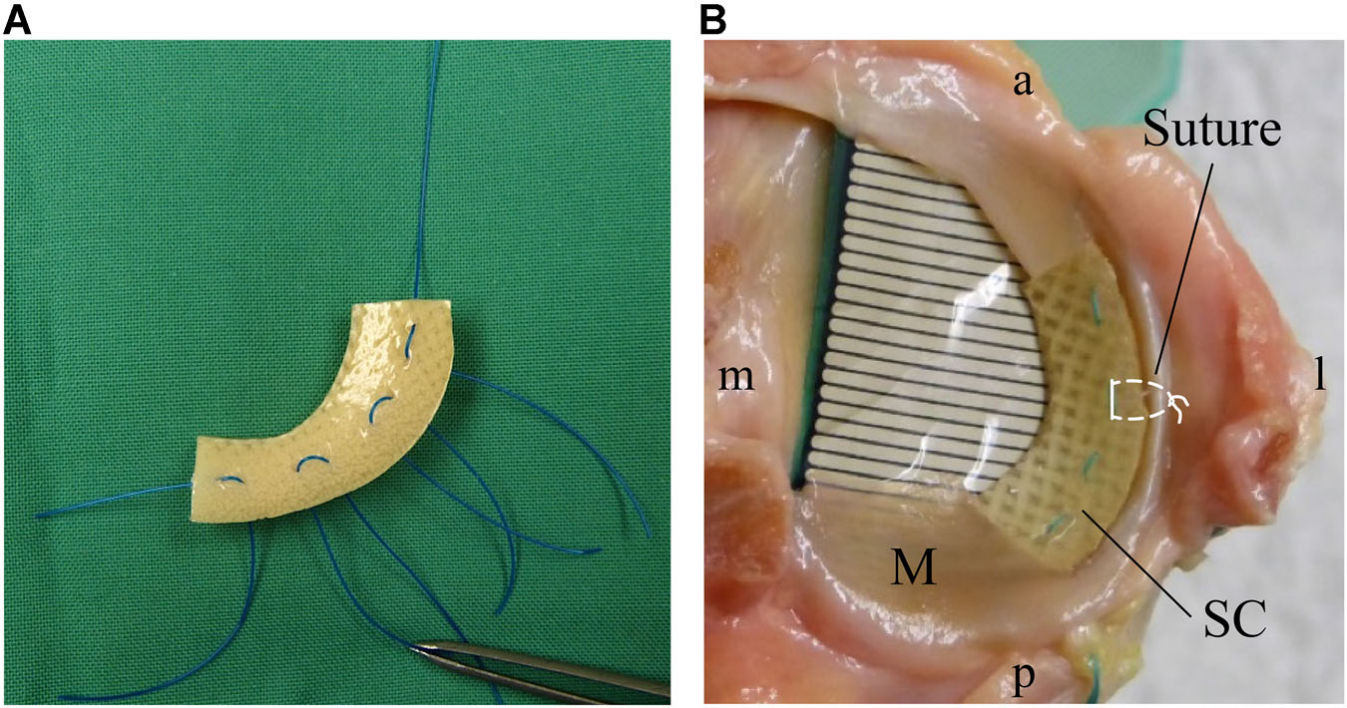
Suture technique for scaffold fixation and placement of pressure sensitive films. (A) Each scaffold was fixed to the meniscal rim using four horizontal mattress sutures (white dashed lines in B). (B) Placement of the TekScan™ sensor on the medial tibial plateau. a , anterior; l,  lateral; M, medial meniscus; m, medial; p, posterior; SC, scaffold. [Color figure can be viewed at wileyonlinelibrary.com]

Silk fibroin implants (FibroFix™; Orthox Ltd.) were manufactured from *Bombyx mori* silkworm silk as described previously.^18^ In brief, silk fibroin fibers were dissolved in lithium bromide and processed into a porous matrix. A horizontally running fiber mesh was integrated into the fibroin matrix to facilitate secure surgical anchoring into the peripheral meniscal rim. The silk fibroin scaffold for replacement of the human medial meniscus is available in three different sizes (Table 1). In the current study, sizing was conducted using anteroposterior X‐rays of the utilized knee joints according to McDermott et al.^20^ The resected part of the medial meniscus was used as a template to trim the implant into the correct individual size and shape (Fig. 1).

**Table 1:** Available Sizes of the Silk Fibroin Scaffold and Number of Devices Implanted in the Current Study

Size	Implant Length (mm)	Implant Width (mm)	Used in the Current Study (*n*)
Small (S)	41.0	8.3	–
Medium (M)	47.2	9.6	5
Large (L)	54.3	11.0	2

### Human Knee Joint Samples and Preparation

This study was approved by the ethics commission of Ulm University (registration number: 206/2016). Seven human knee joints from donors without any medical history of degenerative joint disease (average body mass index 27 kg/m^2^; age: 31–69 y; Science Care, Phoenix, AZ) were prepared and the skin and soft tissues were removed leaving the joint capsule, patellar tendon, and primary stabilizing ligaments intact. The fibula was cut to a length of about 2 cm and fixed to the tibial bone using a cortical screw. Subsequently the bony ends of femur and tibia were fixed in metal pots with polymethyl methacrylate (Technovit 3040; Heraeus Kulzer GmbH, Wehrheim, Germany). Repeated access to the medial meniscus was achieved by a medial parapatellar arthrotomy including an osteotomy of the femoral attachment (*epicondylus medialis*) of the medial collateral ligament. For the sub‐meniscal insertion of the pressure sensitive films, the medial joint capsule as well as the coronary ligament were partially removed. The pressure sensors were fixed to the tibia using two cortical screws to ensure consistent placement. Subsequently, the *epicondylus medialis* was reattached to the femur using a bicortical screw and a claw plate. To ensure defined starting conditions, the patellar tendon was equipped with a woven suture (0.45 mm/20 kg, Flexonit; Cebbra GmbH, Plochingen, Germany) and a 10 N weight, simulating a slightly tensed quadriceps muscle, was attached during testing in the knee joint simulator. After the first measuring cycle with intact meniscus, the medial meniscus was exposed again and a partial meniscectomy was performed leaving the anterior and posterior horns and a 4 mm meniscal rim intact. For the final test, partial meniscal replacement was performed using the silk fibroin implant. The implant was fixed to the meniscal rim using four horizontal mattress sutures (Ethibond EXCEL 3/0; Ethicon, Norderstedt, Germany), which were tied at the meniscus periphery (Fig. 1). During testing the knee joints were kept moist using saline solution.

### Measurement of the Pressure Distribution on the Tibial Plateau

#### Pressure sensitive films and calibration

Contact pressure on the tibial plateau was measured using calibrated pressure sensitive films (Pressure Mapping Sensor Type 4000, 2 × 920.7 mm^2^, 9,000 psi; Tekscan™, Inc., South Boston, MA) (Fig. 1B). The pressure sensors were calibrated using a standard materials testing machine (Z010; Zwick GmbH & Co.KG, Ulm, Germany) and a customized calibration algorithm. 14 pressure values between 0.1 and 8 MPa were applied and calibration was achieved by approximating the obtained raw data (raw sum) in a custom made MATLAB program (MathWorks®, Natick, MA) using a second degree polynomial. Sensors were kept in saline solution 48 h before calibration to ensure the same environmental conditions during calibration as well as during subsequent testing in the knee joint.

#### Knee joint simulator

A previously developed knee joint simulator allowed unconstrained loading of the knee joints under continuous movements of flexion and extension.^19^, ^21^ The femur was fixed in a leverarm, which was moved by an electric motor and the tibia was mounted in a cardan joint, providing all degrees of freedom. The joint position in the simulator was continuously recorded during testing using goniometers. Tibiofemoral and meniscotibial CP was continuously recorded at a constant sample rate of 20 Hz. The testing protocol included two different loading schemes:

(1) Axial load of 200 N.
(2) Axial load of 200 N + external tibial rotation moment (1 Nm).

For each loading scheme, three motion cycles from 0° extension to 100° flexion were performed, thereby, the third cycle was always used for analysis. A custom made MATLAB program was used to calculate CA, maximum pressure on the tibiofemoral CA (CP_max_Cartilage_) as well as maximum pressure on the meniscotibial CA (CP_max_Meniscus_). Therefore, the tibiofemoral as well as meniscotibial CA had to be determined previously. A recording of the pressure sensor was projected onto an image of the tibia plateau using Photoshop CS 4 (Adobe Systems, San José, CA) and a variable rectangle was used to determine the CA between femur and tibia as well as between meniscus and tibia, respectively. Within these rectangles, an area of 3 × 3 sensels was selected, displaying the highest mean pressure which was defined as peak pressure. Finally, for each of the three evaluated parameters (CA, CP_max_Cartilage_, CP_max_Meniscus_) the highest value occurring during a complete flexion and extension cycle was determined.

#### Materials testing machine

During walking, average peak forces of 3.1 times body weight can occur in the human knee joint.^5^ However, the maximum axial load to be applied in the knee joint simulator was limited to 200 N. To evaluate contact mechanics under higher loads, a standard materials testing machine (Z010; Zwick GmbH & Co. KG) was used and axial forces up to 2,500 N (approximately three times body weight) were applied. The knee joints were consecutively tested at four different flexion angles (0°, 30°, 60°, and 90°).

**Figure 2:**
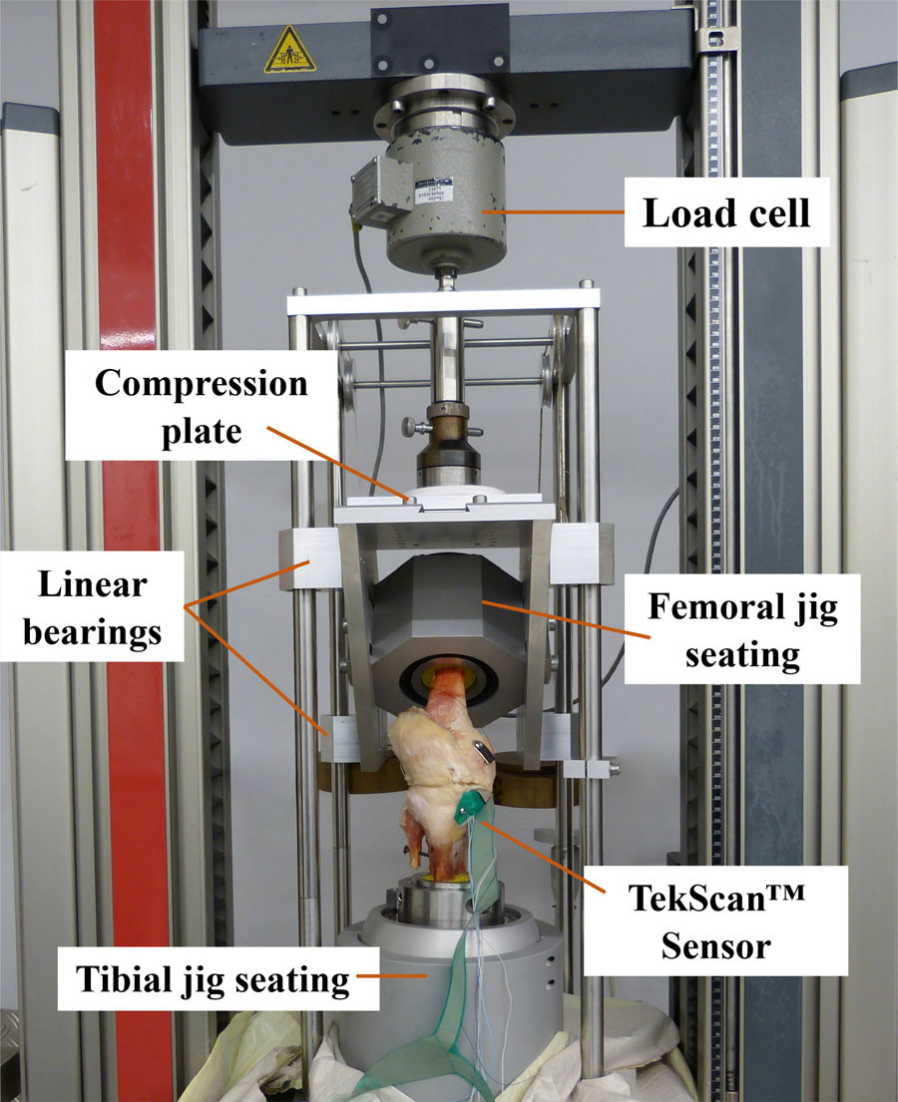
Test setup in the materials testing machine to evaluate contact mechanics (contact area and pressure) under higher loads. The testing rig allowed unconstrained testing of the specimens. The tibial jig seating was placed on a ball bearing panel (not visible) to avoid lateral forces. [Color figure can be viewed at wileyonlinelibrary.com]

The test set up comprised a compression plate connected to a 10 kN load cell (U1; HBM GmbH, Darmstadt, Germany) as well as customized testing rig, which was previously described by Freutel et al.^22^ (Fig. 2). The tibia was mounted in the tibial jig with an integrated ball joint placed on a ball bearing panel, providing all degrees of freedom. Thus, the rig allowed unconstrained testing of the specimens. After applying preloads of 50 N at a constant velocity of 2 mm/min, samples were loaded from 500 to 2,500 N with increments of 500 N. Each load level was held for 5 s. Contact mechanics were analysed at the outset of each load level using MATLAB and the CA, maximum pressure on the tibiofemoral CA (CP_max_Cartilage_) as well as maximum pressure on the meniscotibial CA (CP_max_Meniscus_) were calculated.

### Statistics

Data were presented as mean ± standard deviation and all statistical analyses were performed using Graph Pad Prism (Graph Pad Software Inc., La Jolla, CA).

Results of the continuous motion cycles in the knee joint simulator (CA, CP_max_Cartilage_, CP_max_Meniscus_) were analysed using a one‐way analysis of variance (ANOVA) with post hoc Tukey's test. Different treatments (intact meniscus, meniscectomy, and meniscal replacement) were compared for each loading scheme. Results of the static evaluation in the materials testing machine (CA, CP_max_Cartilage_, CP_max_Meniscus_) were analysed using a two‐way ANOVA with post hoc Tukey's test. Different treatments were compared for each load level at different flexion angles. The level of significance was always set to *p* ≤ 0.05.

## RESULTS

### Tibiofemoral Contact Mechanics Under Continuous Movements of Flexion and Extension

#### CA

**Figure 3:**
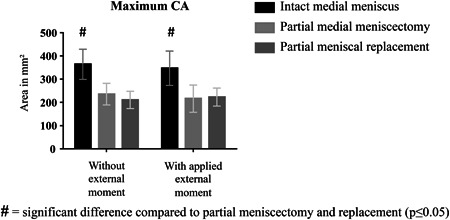
Maximum contact area (CA) in mm^2^ measured on the medial tibial plateau during testing under continuous movements of flexion and extension with and without applied external tibial rotation moments of 1Nm. Mean±standard deviation.

Under an axial load of 200 N, the maximum CA measured during a flexion–extension cycle was significantly reduced by 35.5% after partial meniscectomy compared with the maximum CA with intact meniscus (235.3 ± 46.4 vs. 364.8 ± 64.8 mm^2^; *p* ≤ 0.05). CA was not increased after meniscal replacement, being limited to the area under the silk fibroin implant (210.5 ± 37.4 mm^2^) (Figs. 3 and 5). This was also true when an additional tibial external rotational moment of 1 Nm was applied (intact meniscus: 347.1 ± 73.9 mm^2^ vs. partial meniscectomy: 216.4 ± 58.6 mm^2^ and meniscal replacement: 222.6 ± 38.6 mm^2^; *p* ≤ 0.05).

#### Maximum tibiofemoral CP

**Figure 4:**
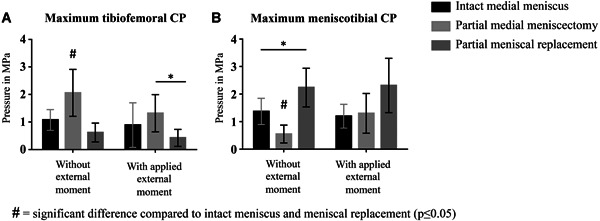
Maximum contact pressure (CP) in MPa measured on the medial tibial plateau during testing under continuous movements of flexion and extension with and without applied external moments. (A) at the tibiofemoral contact zone (B) underneath the medial meniscus. Mean ± standard deviation. *p ≤ 0.05.

Maximum CP between femur and tibial cartilage (CP_max_Cartilage_) during flexion and extension under pure axial loads was 1.1 ± 0.4 MPa. After meniscectomy, pressure on the tibial cartilage was doubled (2.1 ± 0.9 MPa; *p* ≤ 0.05). The subsequent implantation of the silk fibroin implant led to a significant reduction of maximum CP (0.6 ± 0.3 MPa; *p* ≤ 0.05) (Fig. 4A). When an external rotational moment was applied, highest pressure values occurred after partial meniscectomy (1.3±0.7 MPa). After implantation of the silk fibroin implant, pressure was reduced (0.4±0.3 MPa; p ≤ 0.05), displaying even lower pressure values compared with the intact condition (0.9±0.8 MPa) (Fig. 4A). In general, the maximum tibiofemoral CP was always measured at high flexion angles (70°–100°).

#### Maximum meniscotibial CP

**Figure 5:**
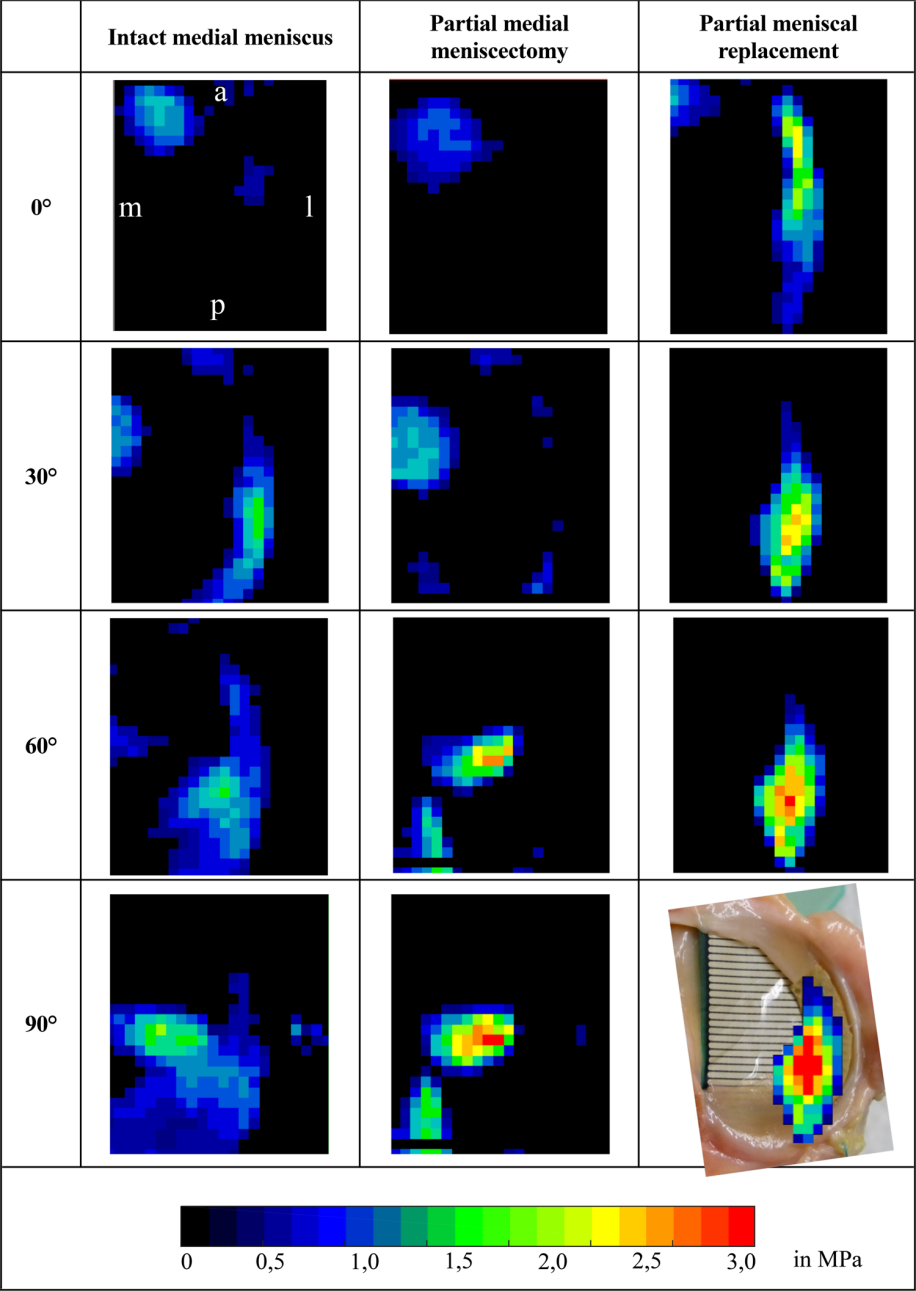
Exemplary recordings of the TekScan™ sensor for the evaluation of the pressure distribution on the medial tibial plateau during dynamic testing in the knee joint simulator under 200 N axial load. a, anterior; l, lateral; m, medial; p, posterior. [Color figure can be viewed at wileyonlinelibrary.com]

Accordingly, maximum CP under the meniscus (CP_max_Meniscus_) was significantly decreased after partial meniscectomy compared with the intact condition (0.5 ± 0.3 vs. 1.4 ± 0.5 MPa; *p* ≤ 0.05). The implantation of the silk fibroin implant shifted the CA to the meniscal replacement device (Fig. 5) leading to a significantly increased meniscotibial pressure (2.2 ± 0.7 MPa; *p* ≤ 0.05) (Fig. 4B). This was also true, when an additional external rotational moment was applied, although there were no significant differences between the different meniscal conditions (intact meniscus: 1.2 ± 0.4 MPa vs. meniscectomy: 1.3 ± 0.7 MPa vs. replacement: 2.3 ± 1.0 MPa) (Fig. 4B). The maximum meniscotibial CP was always measured at high flexion angles between 70° and 100°.

#### Pressure distribution

To analyse the pressure distribution on the tibial plateau, recordings of the pressure mapping sensors at four flexion angles (0°, 30°, 60°, and 90°) were selected. At high flexion angles loading was concentrated at posterior joint regions (Fig. 5). The largest CA was detected with intact meniscus, thereby the axial load was equally distributed over the whole meniscus body at 30°, 60°, and 90°. Consequently, pressure peaks on the articular cartilage were prevented. After partial meniscectomy, CA was considerably reduced and the axial load was mainly transferred through the articular cartilage (CP_max_Cartilage_). The implantation of the silk fibroin scaffold shifted the CA almost completely to the meniscal replacement device rather than distributing the joint force over the complete medial tibial plateau. Hence, the pressure distribution between the intact condition and partial meniscal replacement was considerably different.

### Tibiofemoral Contact Mechanics Under High Axial Loads

Pressure distribution was analysed under incremental axial loads of up 2,500 N (500, 1,000, 1,500, 2,000, 2,500 N). However, as results were comparable for the different load levels, only the results for 2,000 N axial load were presented, as this represents physiological loading occurring during most activities of daily living (225% body weight).^23^

#### CA

**Figure 6:**
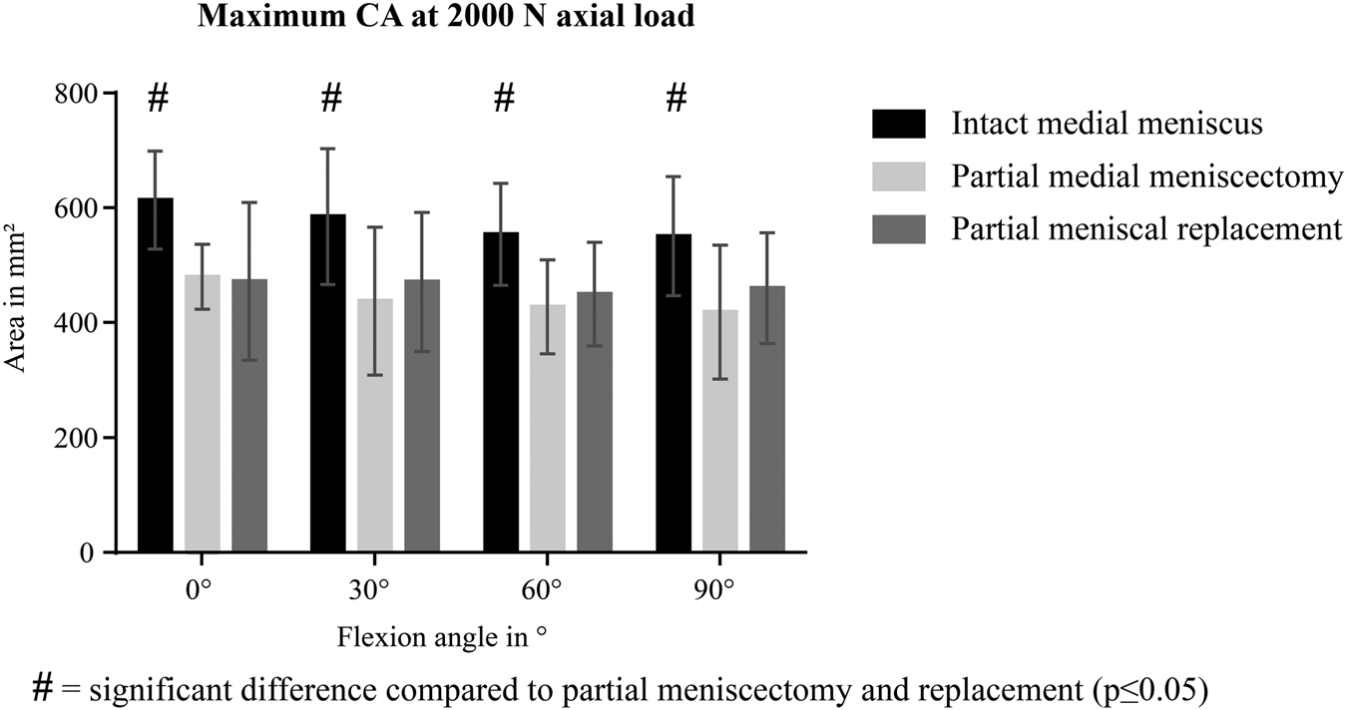
Maximum contact area (CA) in mm^2^ measured on the medial tibial plateau during static testing at 2,000 N axial load. Mean ± standard deviation.

CA between femur and tibia was significantly decreased after meniscectomy compared with the intact condition (*p* ≤ 0.05) (Fig. 6). This was true for all flexion angles. Consistent to the knee simulator experiments, the subsequent implantation of the silk fibroin scaffold could not increase the reduced CA. With intact meniscus, CA was largest in full extension (0°) and decreased slightly with increasing flexion angle. This shift was less pronounced after meniscectomy and scaffold implantation.

#### Maximum tibiofemoral CP

**Figure 7:**
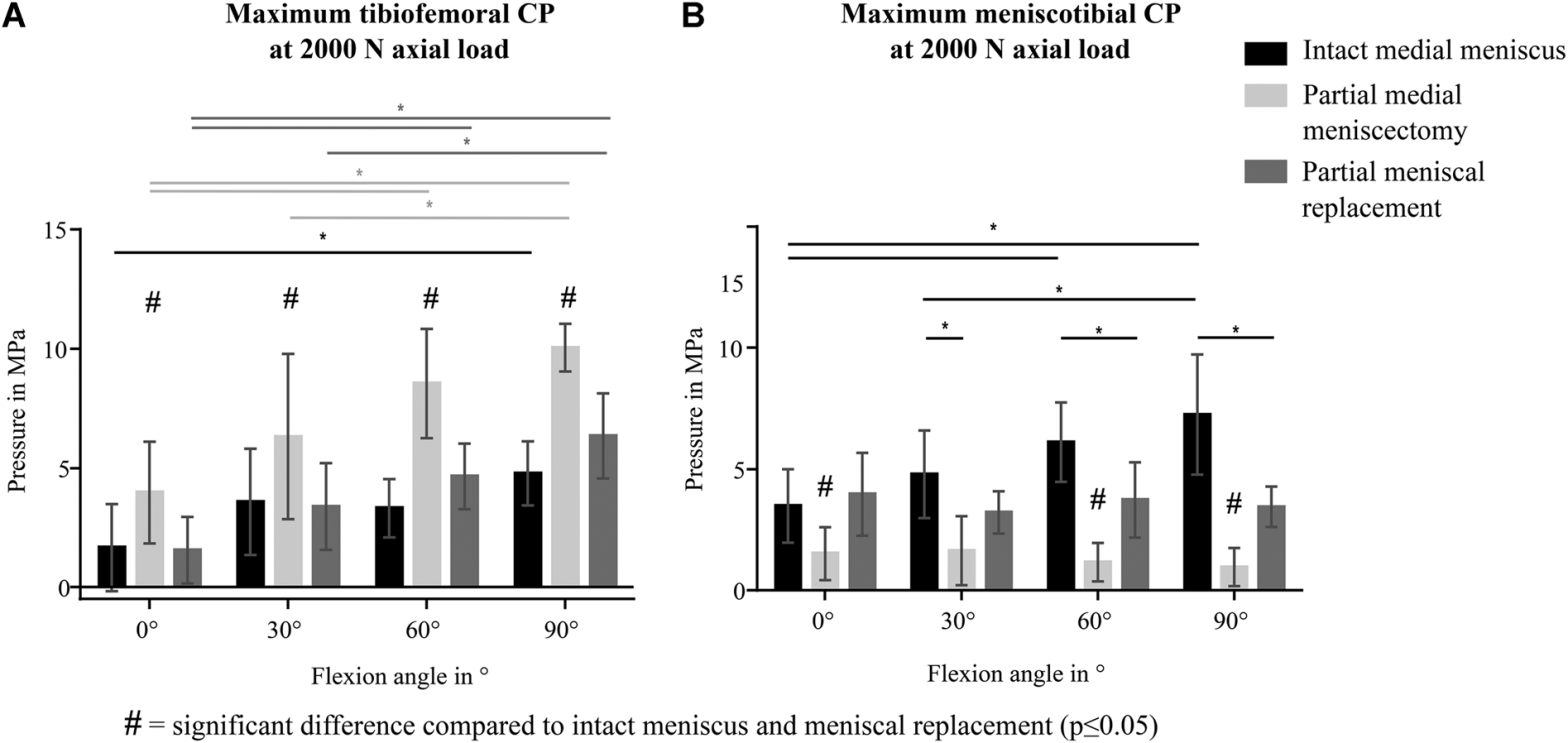
Maximum contact pressure (CP) in MPa measured on the medial tibial plateau during static testing at 2,000 N axial load. (A) at the tibiofemoral contact zone (B) underneath the medial meniscus. *p ≤ 0.05. Mean ± standard deviation.

Maximum CP between femoral and tibial cartilage (CP_max_Cartilage_) was highest after partial meniscectomy compared with the intact meniscus condition as well as scaffold implantation (Fig. 7A). Thereby, CP_max_Cartilage_ was more than doubled after meniscectomy. This was true for all flexion angles, thereby, maximum pressure increased with increasing flexion angles. After scaffold implantation, maximum CP was restored to the intact condition at 0° and 30° flexion.

#### Maximum meniscotibial CP

The maximum CP measured between meniscus or scaffold and tibial cartilage (CP_max_Meniscus_), was lowest after partial meniscectomy, showing significant differences to the intact meniscus as well as scaffold implantation at 0°, 60°, and 90° of flexion (Fig. 7B). Although meniscal replacement was able to increase meniscotibial CP after meniscectomy, it remained below the pressure values of the intact condition at 60° and 90° flexion (*p* ≤ 0.05). With intact meniscus, pressure values increased with increasing flexion angle. At 90° flexion, CP was twice as high compared with the maximum pressure in full extension (0°).

#### Pressure distribution

**Figure 8:**
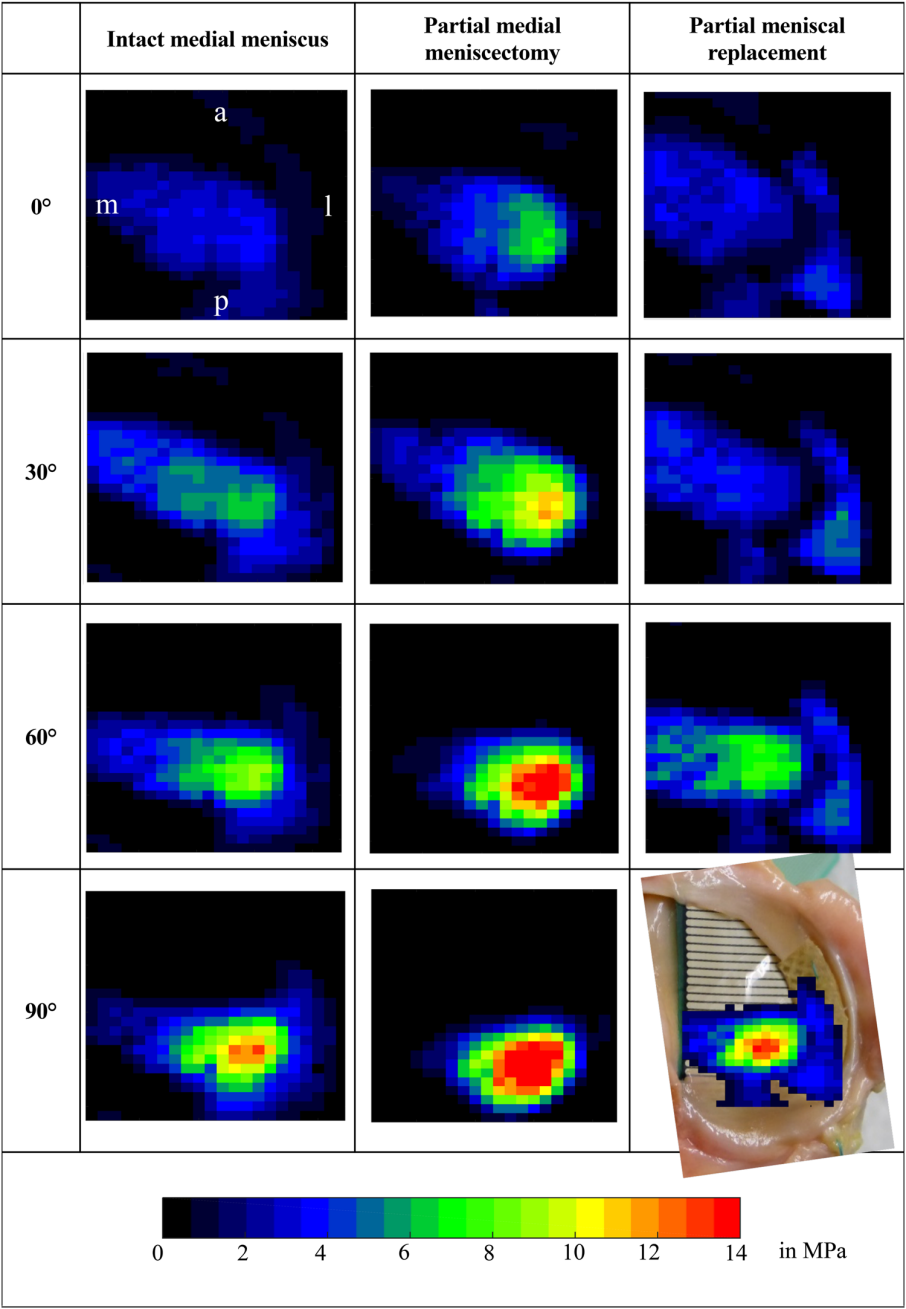
Exemplary recordings of the TekScan™ sensor for the evaluation of the pressure distribution on the medial tibial plateau during static testing in the materials testing machine under 2,000 N axial load. a, anterior; l, lateral; m, medial; p, posterior. [Color figure can be viewed at wileyonlinelibrary.com]

Recordings of the Tekscan™ sensors showed that at all flexion angles, largest CA was detected with intact meniscus, confirming measurements of the knee joint simulator. With intact meniscus, CP was mainly transmitted through the meniscus (CP_max_Meniscus_) and evenly distributed, thereby avoiding pressure peaks on the tibial cartilage (Fig. 8). With increasing flexion angles, CA decreased and CP occurred more posteriorly. After partial meniscectomy, CA was decreased and consequently tibiofemoral CP (CP_max___Cartilage_) increased. Similar to the intact condition, pressure was applied more posteriorly with the knee flexed. After meniscal replacement, pressure distribution was similar to the intact condition at 0° and 30° of flexion and CP was mainly transmitted through the implant. Especially at high flexion angles, tibial cartilage was heavily loaded. Furthermore, pressure was not distributed over the remaining meniscal tissue.

## DISCUSSION

The most important finding of the current study was that partial meniscal replacement using the silk fibroin scaffold considerably improved disturbed tibiofemoral contact mechanics after partial medial meniscectomy. However, it was not possible to completely restore physiological knee joint pressure distribution comparable to the intact condition. Thus, neither under continuous movements of flexion and extension, nor under static but physiologically high loaded test conditions, the reduced CA after meniscectomy could be restored by implantation of the silk fibroin scaffold. Clinically relevant peak pressures on the articular cartilage surface (CP_max___Cartilage_) occurring after partial meniscectomy were significantly reduced by meniscal replacement. However, static testing at high flexion angles revealed higher peak pressures on the cartilage surface after implantation of the scaffold compared with the intact condition. The reduced maximum CP under the meniscus in the partially meniscectomised joint was increased by meniscal replacement. Nevertheless, static testing at high flexion angles demonstrated that normal pressure distribution comparable to the intact meniscus could not be achieved.

Previous studies, investigating tibiofemoral contact mechanics after implantation of different materials for meniscal replacement, have also failed to demonstrate a complete restoration of a reduced CA seen after partial or total meniscectomy.^24^, ^25^ Brophy et al.^25^ tested a porous polyurethane scaffold for partial lateral meniscal replacement in a defect of similar size as in the current study. In accordance with the current study, there were significantly decreased average and peak CPs after scaffold implantation compared with partial meniscectomy while CA could not be completely restored to the intact condition. Vrancken et al.^24^ investigated a polycarbonate urethane (PCU) implant for total meniscus replacement in a knee loading rig under 1,000 N axial load. Thereby, mean and peak CPs were significantly increased after meniscus replacement. Although tibiofemoral CA was slightly increased compared with the meniscectomised joint, there was a significant reduction compared with the native meniscus condition. Consequently, the PCU device was not able to restore physiological knee joint contact mechanics and the authors concluded that an inadequate fixation strategy might be a possible reason.

For the silk fibroin scaffold evaluated in the current study, inadequate size matching might be a reason for the lack of improvement of CA after implantation. Given the high natural variability of anatomical structures,^26^ optimal adaption to the meniscal host tissue was not always possible as the scaffold was only available in three different sizes for human use. Visible differences in height have partially led to an incongruity over the meniscus‐implant‐surface and consequently insufficient distribution of load over the whole meniscus‐implant construct. As the implanted scaffold was thicker than the surrounding meniscal tissue, only the implant was loaded during axial compression and the resulting CA was considerably reduced compared with the intact situation. Future manufacturing of patient specific implants or a wider range of scaffold sizes, might improve implant adaption to the host tissue.

It was previously shown that degenerative changes of the cartilage surface initially occur in weight‐bearing areas of direct tibiofemoral contact as peak pressures are especially high in these locations.^12^, ^27^ Accordingly, Brophy et al.^25^ could show that partial meniscectomy leads to an increase in Cs close to the *eminentia intercondylaris*. Tibiofemoral contact mechanics under different meniscal conditions were previously investigated using different test setups.^11^, ^12^, ^28^, ^29^, ^30^, ^31^, ^32^ However, to the best of our knowledge, there is only one study in the literature differentiating local peak contact pressures on the tibial plateau (tibiofemoral vs. meniscotibial pressures) when evaluating the effect of meniscal replacement on intra‐articular contact mechanics.^31^ In this study by Paletta et al., peak pressures increased considerably after total lateral meniscectomy, which coincides with the current study. Subsequent meniscal allograft implantation reduced peak pressures at the tibiofemoral CA, however peak pressures were up to 86% higher compared with the intact condition. Furthermore, peak meniscotibial pressures could also not be restored after meniscal replacement and remained up to 38% below the values measured for the intact condition. Clinically, it is known that meniscal allograft implantation reduces pain and improves knee joint function during daily activities.^33^ Therefore, it can be speculated that it might not be necessary to completely restore knee joint contact mechanics after meniscal replacement in order to provide clinical benefit.^24^ The silk fibroin scaffold evaluated in the current study also failed to completely restore physiological peak pressures. Under an axial load of 2,000 N and at low flexion angles, peak contact pressures under the meniscus as well as at the tibiofemoral contact zone were restored after scaffold implantation. However, at 60° and 90° of flexion, meniscotibial peak pressures were significantly lower compared with the intact meniscus condition. Accordingly, tibiofemoral pressure was also higher for these flexion angles compared with the intact condition, indicating persistent overloading of the articulating cartilage surfaces despite meniscal substitution. Under continuous movements of flexion and extension and an applied load of only 200 N, tibiofemoral peak pressure in the meniscectomised joint was considerably reduced after meniscal replacement. However, meniscotibial peak contact pressure was about twice as high compared with the intact condition. The reason might be that under an applied load of only 200 N, most of the load was transferred through the scaffold, probably due to its previously demonstrated high stiffness.^18^ This effect was not present under high loads of 2,000 N, when the implant was more compressed and consequently loading was more pronounced on the tibiofemoral cartilage in areas close to the joint midline. This emphasizes the importance of testing under physiological high joint loads.

Our formulated hypothesis that partial meniscal replacement using the silk fibroin scaffold will restore tibiofemoral contact mechanics to the intact situation could only partially be confirmed. Similar to previous studies investigating tibial contact mechanics after meniscus replacement, the reduced CA after meniscectomy was not improved.^24^, ^25^ Furthermore, physiological pressure distributions over the whole range of motion comparable to the intact condition were not achieved. This emphasizes the huge challenge of optimal meniscal substitution in the complex loading situation of the knee joint. Despite extensive research in this field over the recent years, none of the materials tested in similar setups were able to entirely compensate for native meniscus function over the full range of loading and flexion scenarios tested. However, this failure to fully replicate meniscal contact pressures is also observed in meniscal allograft transplantation in which clinical utility has been demonstrated indicating that complete restoration of meniscal biomechanics may not be a pre‐requisite for improved patient knee function. This is particularly the case when considering the improved performance of meniscal substitute materials in these studies over the current standard of care, meniscectomy. As the silk fibroin implant was not adequately adapted into the height of the meniscal defects created in this study, the device was not optimized for redistribution of loads over the complete meniscus‐implant‐surface. Especially at high flexion angles, cartilage was still heavily loaded. Future improvements according to the requirements for meniscal substitution postulated by Rongen et al.^13^ are necessary to completely restore tibiofemoral contact mechanics, with patient specific implants designed on the basis of previously acquired magnetic resonance imaging scans being a potential solution to improve implant fit.

A limitation of the current study was that the investigation of tibiofemoral contact mechanics should ideally be performed under continuous movements of flexion and extension as well as under physiological loads. This was not possible in the current study. However, simulating physiological joint movements under low loads is also justified as rehabilitation after meniscus surgery usually includes a period of partial weight‐bearing.

## CONCLUSION

Partial meniscal replacement using the silk fibroin scaffold considerably improved disturbed tibiofemoral contact mechanics after partial medial meniscectomy. However, it was not possible to completely restore physiological knee joint pressure distribution. Static testing at high flexion angles revealed higher local peak pressures on the cartilage surface after implantation of the scaffold compared with the intact condition. This emphasizes the huge challenge of optimal meniscal substitution in the complex loading situation of the knee joint. Future improvements, according to the postulated requirements for meniscal substitution, are necessary to completely restore tibiofemoral contact mechanics. To achieve this, patient specific implants could considerably improve implant geometry and fixation.

## ACKNOWLEDGMENTS

We would like to thank Steffen Hacker and Nicolas Wolf for their support regarding sensor calibration and data analysis. This work was financially supported by the German Armed Forces [E/U2AD/ED001/EF551] as well as by the Wellcome Trust [100917/z/13/z]. This report is independent research funded by the National Institute for Health Research (Invention for Innovation (i4i), Development of manufacturing capability and pilot clinical evaluation of FibroFix: A mechanically advanced, tissue regenerative, meniscal cartilage repair device, II‐LB‐0417‐20005). The views expressed in this publication are those of the author(s) and not necessarily those of the NHS, the National Institute for Health Research or the Department of Health and Social Care.

## References

[1] Kurosawa H , Fukubayashi T , Nakajima H . 1980. Load‐bearing mode of the knee joint: physical behavior of the knee joint with or without menisci. Clin Orthop Relat Res 149:283–290. 7408313

[2] Seedhom BB . 1976. Loadbearing function of the menisci. Physiotherapy 62:223. 989604

[3] Walker PS , Erkiuan MJ . 1975. The role of the menisci in force transmission across the knee. Clin Orthop Relat Res 109:184–192. 10.1097/00003086-197506000-00027 1173360

[4] Masouros SD , McDermott ID , Amis AA , et al. 2008. Biomechanics of the meniscus‐meniscal ligament construct of the knee. Knee Surg Sports Traumatol Arthrosc 16:1121–1132. 18802689 10.1007/s00167-008-0616-9

[5] Taylor WR , Heller MO , Bergmann G , et al. 2004. Tibio‐femoral loading during human gait and stair climbing. J Orthop Res 22:625–632. 15099644 10.1016/j.orthres.2003.09.003

[6] Peña E , Calvo B , Martínez MA , et al. 2005. Finite element analysis of the effect of meniscal tears and meniscectomies on human knee biomechanics. Clin Biomech 20:498–507. 10.1016/j.clinbiomech.2005.01.009 15836937

[7] Majewski M , Susanne H , Klaus S . 2006. Epidemiology of athletic knee injuries: a 10‐year study. Knee 13:184–188. 16603363 10.1016/j.knee.2006.01.005

[8] Hede A , Larsen E , Sandberg H . 1992. Partial versus total meniscectomy. A prospective, randomised study with long‐term follow‐up. J Bone Joint Surg Br 74:118–121. 1732238 10.1302/0301-620X.74B1.1732238

[9] Roos H , Lauren M , Adalberth T , et al. 1998. Knee osteoarthritis after meniscectomy: prevalence of radiographic changes after twenty‐one years, compared with matched controls. Arthritis Rheum 41:687–693. 9550478 10.1002/1529-0131(199804)41:4<687::AID-ART16>3.0.CO;2-2

[10] Englund M , Lohmander LS . 2004. Risk factors for symptomatic knee osteoarthritis fifteen to twenty‐two years after meniscectomy. Arthritis Rheum 50:2811–2819. 15457449 10.1002/art.20489

[11] Seitz AM , Lubomierski A , Friemert B , et al. 2012. Effect of partial meniscectomy at the medial posterior horn on tibiofemoral contact mechanics and meniscal hoop strains in human knees. J Orthop Res 30:934–942. 22072570 10.1002/jor.22010

[12] Lee SJ , Aadalen KJ , Malaviya P , et al. 2006. Tibiofemoral contact mechanics after serial medial meniscectomies in the human cadaveric knee. Am J Sports Med 34:1334–1344. 16636354 10.1177/0363546506286786

[13] Rongen JJ , van Tienen TG , van Bochove B , et al. 2014. Biomaterials in search of a meniscus substitute. Biomaterials 35:3527–3540. 24477194 10.1016/j.biomaterials.2014.01.017

[14] Stone KR , Steadman JR , Rodkey WG , et al. 1997. Regeneration of meniscal cartilage with use of a collagen scaffold. Analysis of preliminary data. J Bone Joint Surg 79:1770–1777. 9409790 10.2106/00004623-199712000-00002

[15] Kelly BT , Robertson W , Potter HG , et al. 2007. Hydrogel meniscal replacement in the sheep knee: preliminary evaluation of chondroprotective effects. Am J Sports Med 35:43–52. 16957008 10.1177/0363546506292848

[16] Gruchenberg K , Ignatius A , Friemert B , et al. 2015. In vivo performance of a novel silk fibroin scaffold for partial meniscal replacement in a sheep model. Knee Surg Sports Traumatol Arthrosc 23:2218–2229. 24770350 10.1007/s00167-014-3009-2 PMC4661201

[17] Warnecke D , Schild NB , Klose S , et al. 2017. Friction properties of a new silk fibroin scaffold for meniscal replacement. Tribol Int 109:586–592. 28469288 10.1016/j.triboint.2017.01.038 PMC5327953

[18] Warnecke D , Stein S , Haffner‐Luntzer M , et al. 2018. Biomechanical, structural and biological characterisation of a new silk fibroin scaffold for meniscal repair. J Mech Behav Biomed Mater 86:314–324. 30006280 10.1016/j.jmbbm.2018.06.041 PMC6079190

[19] Dürselen L , Claes L , Kiefer H . 1995. The influence of muscle forces and external loads on cruciate ligament strain. Am J Sports Med 23:129–136. 7726343 10.1177/036354659502300122

[20] McDermott ID , Sharifi F , Bull AMJ , et al. 2004. An anatomical study of meniscal allograft sizing. Knee Surg Sports Traumatol Arthrosc 12:130–135. 12756521 10.1007/s00167-003-0366-7

[21] Dürselen L , Hebisch A , Claes LE , et al. 2003. Gapping phenomenon of longitudinal meniscal tears. Clin Biomech 18:505–510. 10.1016/s0268-0033(03)00057-3 12828899

[22] Freutel M , Seitz AM , Ignatius A , et al. 2015. Influence of partial meniscectomy on attachment forces, superficial strain and contact mechanics in porcine knee joints. Knee Surg Sports Traumatol Arthrosc 23:74–82. 24671386 10.1007/s00167-014-2951-3

[23] Kutzner I , Heinlein B , Graichen F , et al. 2010. Loading of the knee joint during activities of daily living measured in vivo in five subjects. J Biomech 43:2164–2173. 20537336 10.1016/j.jbiomech.2010.03.046

[24] Vrancken AC , Eggermont F , van Tienen TG , et al. 2016. Functional biomechanical performance of a novel anatomically shaped polycarbonate urethane total meniscus replacement. Knee Surg Sports Traumatol Arthrosc 24:1485–1494. 25971457 10.1007/s00167-015-3632-6 PMC4853448

[25] Brophy RH , Cottrell J , Rodeo SA , et al. 2010. Implantation of a synthetic meniscal scaffold improves joint contact mechanics in a partial meniscectomy cadaver model. J Biomed Mater Res Part A 92:1154–1161. 10.1002/jbm.a.32384 19322823

[26] Vrancken AC , Crijns SP , Ploegmakers MJ , et al. 2014. 3D geometry analysis of the medial meniscus—a statistical shape modeling approach. J Anat. 225:395–402. 25052030 10.1111/joa.12223 PMC4174023

[27] Korkala O , Karaharju E , Grönblad M , et al. 1984. Articular cartilage after meniscectomy: rabbit knees studied with the scanning electron microscope. Acta Orthop Scand 55:273–277. 6547558 10.3109/17453678408992355

[28] Baratz ME , Fu FH , Mengato R . 1986. Meniscal tears: the effect of meniscectomy and of repair on intraarticular contact areas and stress in the human knee: a preliminary report. Am J Sports Med 14:270–275. 3755296 10.1177/036354658601400405

[29] Fukubayashi T , Kurosawa H . 1980. The contact area and pressure distribution pattern of the knee: a study of normal and osteoarthrotic knee joints. Acta Orthop Scand 51:871–879. 6894212 10.3109/17453678008990887

[30] Ahmed AM , Burke DL . 1983. In‐vitro of measurement of static pressure distribution in synovial joints—Part I: tibial surface of the knee. J Biomech Eng 105:216–225. 6688842 10.1115/1.3138409

[31] Paletta GA Jr. , Manning T , Snell E , et al. 1997. The effect of allograft meniscal replacement on intraarticular contact area and pressures in the human knee: a biomechanical study. Am J Sports Med 25:692–698. 9302479 10.1177/036354659702500519

[32] Ihn JC , Kim SJ , Park IH . 1993. In vitro study of contact area and pressure distribution in the human knee after partial and total meniscectomy. Int Orthop 17:214–218. 8407035 10.1007/BF00194181

[33] Crook T , Ardolino A , Williams L , et al. 2009. Meniscal allograft transplantation: a review of the current literature. Ann R Coll Surg Engl 91:361–365. 19409145 10.1308/003588409X428559 PMC2758427

